# The neutrophil percentage-to-albumin ratio is an independent risk factor for poor prognosis in peritoneal dialysis patients

**DOI:** 10.1080/0886022X.2023.2294149

**Published:** 2024-01-04

**Authors:** Mingfan Xu, Jingjia Huan, Lujie Zhu, Jiachun Xu, Kai Song

**Affiliations:** Department of Nephrology, The Second Affiliated Hospital of Soochow University China, China

**Keywords:** Peritoneal dialysis, neutrophil, serum albumin, percentage-to-albumin ratio (NPAR;), prognosis

## Abstract

**Aim:**

This study aimed to investigate the predictive ability of the neutrophil percentage-to-albumin Ratio (NPAR) concerning all-cause mortality and cardio-cerebrovascular mortality in patients undergoing peritoneal dialysis (PD).

**Methods:**

We included a total of 807 PD patients from the Peritoneal Dialysis Center of the Second Affiliated Hospital of Soochow University between January 2009 and December 2019 in this study. Patients were categorized into three groups based on their baseline NPAR. The Kaplan-Meier method, multivariate Cox proportional hazard model, and Fine-Gray competing risk model were employed to examine the relationship between NPAR level and all-cause mortality and cardio-cerebrovascular mortality among PD patients. Furthermore, the ROC curve and calibration plots were utilized to compare the performance between NPAR and other conventional indicators.

**Results:**

The mean follow-up period was 38.2 months. A total of 243 (30.1%) patients passed away, with 128 (52.7%) succumbing to cardio-cerebrovascular diseases. The mortality rates of the Middle and High NPAR groups were significantly greater than that of the Low NPAR group (*p* < 0.001), and NPAR was independently associated with all-cause mortality and cardio-cerebrovascular mortality. Receiver Operating Characteristic (ROC) analysis indicated that the Area Under the Curve (AUC) of NPAR (0.714) was significantly superior to those of C-reactive protein (CRP) (0.597), neutrophil to lymphocyte ratio (NLR) (0.589), C-reactive protein to albumin ratio (CAR) (0.698) and platelet to lymphocyte ratio (PLR) (0.533).

**Conclusion:**

NPAR served as an independent predictive marker for all-cause mortality and cardio-cerebrovascular mortality in PD patients. Moreover, NPAR demonstrated superior predictive potential compared to CRP, CAR, NLR, and PLR.

## 1. Introduction

Peritoneal dialysis (PD) is one of the renal replacement therapies for end-stage renal disease (ESRD). The mortality of PD patients has been steadily increasing, with cardiovascular death being the primary cause [1–3]. Previous research has demonstrated that chronic inflammation and malnutrition are two major risk factors associated with poor prognosis in PD patients [4,5]. However, the benefit of adjusting these indicators is still unclear. Large-scale prospective studies have shown interventions for some clinical parameters, including anemia, hyperphosphatemia, and oxidative stress do not improve the survival of ESRD patients [6]. Therefore, new biomarkers related to the prognosis of PD, especially some composite markers reflecting multiple pathological processes, need further exploration.

The neutrophil percentage-to-albumin ratio (NPAR), which is the ratio of neutrophil percentage (Neu%) to serum albumin (ALB), is a recently discovered indicator for predicting prognosis in many diseases. For example, increased NPAR has been found to be associated with poor outcomes in many cancer patients [7]. Likewise, high levels of NPAR have been suggested as an independent risk factor for all-cause mortality in patients with cardiovascular diseases [8–10]. However, a lack of research remains on the correlation between the NPAR and mortality in PD patients. Given that cardiovascular disease is the leading cause of death in PD patients, we propose that increased NPAR may also be associated with increased mortality in PD patients. Moreover, as NPAR is a composite parameter reflecting inflammation and malnutrition, it is of interest to assess the predictive value of NPAR compared to C-reactive protein (CRP) and ALB [11], and with other composite parameters associated with the mortality of PD patients, including neutrophil to lymphocyte ratio (NLR)[12], C-reactive protein to albumin ratio (CAR) and platelet to lymphocyte ratio (PLR) [13].

Therefore, this retrospective study aimed to investigate whether the NPAR was associated with all-cause mortality and cardio-cerebrovascular mortality in PD patients. We also compared the predictive value of the NPAR and other indicators, such as CRP, ALB, CAR, NLR and PLR, on mortality in PD patients.

## 2. Materials and methods

### 2.1. Subjects

A total of 930 peritoneal dialysis patients were enrolled from January 2009 to December 2019 at the Peritoneal Dialysis Center of the Second Affiliated Hospital of Soochow University according to the following criteria. Inclusion criteria: (1) ≥18 years of age; (2) who had been on continuous ambulatory peritoneal dialysis for at least 3 months; (3) who had at least one visit to the dialysis center and could provide biochemical data; and (4) who had recorded their endpoints during follow-up. Exclusion criteria: (1) patients with malignant tumors or other serious organ dysfunction affecting inflammatory marker levels; (2) patients with peritonitis or other infections in the past month; and (3) patients with no follow-up data or unable to obtain laboratory parameters.

### 2.2. Data collection

Clinical data were collected, including sex, age, and comorbidities [hypertension, diabetes, cardiovascular disease (CVD)]. CVD was referred to as myocardial infarction, pericarditis, myocarditis, coronary artery atherosclerotic heart disease, arrhythmia, valvular heart disease, heart failure, etc. Laboratory data were collected on the third month of stable dialysis in patients, including white blood cells, neutrophils, lymphocytes, NLR, monocytes, hemoglobin, platelets, creatinine, urea nitrogen, uric acid, corrected calcium, phosphorus, albumin, prealbumin, CRP, CAR, intact parathyroid hormone (iPTH), total bilirubin, total cholesterol, triglycerides, low-density lipoprotein, high-density lipoprotein, blood glucose, and blood potassium. All cases with missing data and outliers were removed from the cohort.

### 2.3. Endpoints

Patients were followed by telephone, outpatient review or home visits until 31 December 2019. Endpoints included peritoneal dialysis status, all-cause death, cardio-cerebrovascular death, and termination of peritoneal dialysis (such as transfer to hemodialysis, kidney transplantation, and improvement of primary kidney disease).

### 2.4. Methods

The data were analyzed using SPSS 26.0 and R 4.22 statistical software. Normally distributed data are expressed as the mean ± standard deviation (SD), and nonnormally distributed data are presented as the median with interquartile range (IQR). Quantitative data were analyzed using variance analysis for normally distributed data and the Kruskal–Wallis H rank test for nonnormally distributed data. Categorical data were compared between groups using Pearson’s χ^2^ test. Kaplan–Meier survival curves and log-rank tests were used to compare survival rates between groups. The Cox proportional hazards model was used to investigate the association between the NPAR and all-cause mortality among PD patients. The dependent variable in our analysis was time-to-event, where the event was defined as death from any cause. Patients who were alive at the last follow-up or at the end of the study were treated as censored observations. The primary independent variable was the NPAR, and the covariates were age, sex, hypertension, diabetes, cardiovascular disease, hemoglobin, platelets, serum creatinine, phosphorus, low density lipoprotein and C-reactive protein. We checked the proportional hazards assumption for each covariate by the Schoenfeld Residual Test (Supplementary Figure s1), and no significant violation was found except for hypertension. However, based on clinical relevance, we still included it as a covariate. The Fine-Grey competing risk model was used to analyze the risk factors for cardio-cerebrovascular death across NPAR groups. ROC curves for single factors were used to compare the predictive ability of the NPAR and traditional prognostic indicators for the outcome of PD patients. Calibration plots were generated to assess the agreement between the predicted probabilities from our regression model and the observed outcomes of all-cause mortality in PD patients. A perfectly calibrated model would result in points lying along a 45-degree diagonal line. All significance levels were set at *p* < 0.05.

## 3. Results

### 3.1. Baseline characteristics

**Figure 1.**
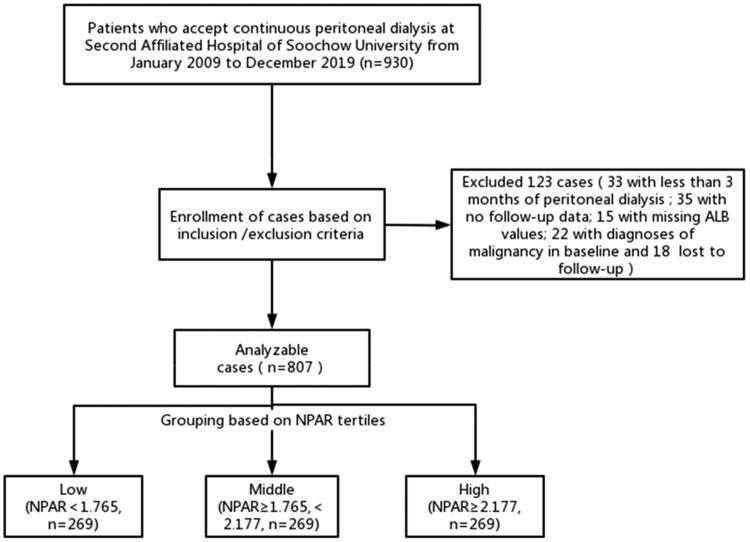
Flowchart of study population. Abbreviation: ALB: albumin; NPAR: neutrophil percentage-to-albumin ratio.

A total of 930 PD patients in the Peritoneal Dialysis Center of the Second Affiliated Hospital of Soochow University from 2009 to 2019 were included in the study, 123 of whom were excluded, including 33 with peritoneal dialysis less than 3 months, 35 without follow-up data, 15 missing ALB values, 22 with malignancy at the start of PD, and 18 lost during the follow-up, leaving 807 patients (Figure 1). The average age of the selected patients was 60.0 ± 16.0 years, 56.9% of whom were male, 89.9% had hypertension, 29.0% had diabetes, and 22.7% had CVD. According to the baseline NPAR value, the patients were divided into low (NPAR <1.765, *n* = 269), middle (NPAR ≥1.765, <2.177, *n* = 269), and high (NPAR ≥2.177, *n* = 269) NPAR groups. Compared with the low NPAR group, the high NPAR group had a shorter survival time, older age, higher proportions of diabetes and cardiovascular diseases, and higher levels of inflammatory-related parameters such as WBC, CRP, CAR, and NLR (*p* < 0.05). The proportion of hypertension was similar among the three groups (*p* = 0.987) (Table 1).

**Table 1.** Baseline characteristics across different NPAR groups.

Factors	Low (<1.765, *n* = 269)	Middle (≥1.756, <2.177, *n* = 269)	High (≥2.177, *n* = 269)	Total	*p*-value
Age (year)^a^	56.2 ± 16.4	60.1 ± 15.6	63.5 ± 14.8	60.0 ± 16.0	<0.001
Survive (month)^a^	45.2 (21, 69.5)	35.8 (15.5, 56)	33.8 (12, 55.5)	38.2 (15, 61.5)	<0.001
Male [case (%)]	156 (19.3)	153 (19.0)	150 (18.6)	459 (56.9)	0.872
Diabetes [case (%)]^a^	52 (6.44)	80 (9.91)	102 (12.6)	234 (29.0)	<0.001
Hypertension [case (%)]	242 (30.0)	241 (29.9)	242 (30.0)	725 (89.9)	0.987
CVD [case (%)]	52 (19.3)	60 (7.43)	71 (8.80)	183 (22.7)	0.145
All cause Death [case (%)]^a^	36 (4.46)	76 (9.42)	131 (16.2)	243 (30.1)	<0.001
Cardio-cerebrovascular Death [case (%)]^a^	21 (2.60)	43 (5.33)	64 (7.93)	128 (15.86)	<0.001
WBC (×10^9^g/L)^a^	6.09 ± 1.69	6.37 ± 2.13	7.29 ± 2.80	6.59 ± 2.31	<0.001
Neutrophils (×10^9^g/L)^a^	3.71 ± 1.29	4.32 ± 1.72	5.49 ± 2.55	4.51 ± 2.06	<0.001
Neutrophil Percentage (%)^a^	60.29 ± 8.83	66.90 ± 7.24	73.70 ± 8.60	66.96 ± 9.90	<0.001
Lymphocyte (×10^9^g/L)^a^	1.73 ± 0.57	1.38 ± 0.53	1.17 ± 0.46	1.43 ± 0.57	<0.001
NLR^a^	2.24 (1.67, 2.80)	3.18 (2.45, 3.90)	4.8 (3.20, 6.40)	3.23 (2.18, 4.28)	<0.001
Monocyte (×10^9^g/L)^a^	0.37 ± 0.18	0.40 ± 0.18	0.45 ± 0.27	0.41 ± 0.22	<0.001
Hemoglobin (g/L)^a^	116.9 ± 21.9	113.0 ± 23.3	104.5 ± 23.1	111.5 ± 23.4	<0.001
Platelet (g/L)	179.7 ± 56.2	175.0 ± 69.8	184.3 ± 79.2	179.7 ± 69.0	0.296
PLR	170.5 (130, 211)	169.5 (130, 209)	177 (130, 209)	173 (129, 217)	0.649
SCR (mmol/L)^a^	756 ± 287	776 ± 325	680 ± 306	737 ± 309	0.01
BUN (mmol/L)^a^	18.47 ± 5.40	18.66 ± 6.03	17.42 ± 6.69	18.18 ± 6.08	0.039
Uric acid (mmol/L)^a^	439 ± 99	446 ± 110	412 ± 102	443 ± 105	<0.001
Ca^2+^ (mmol/L)^a^	2.24 ± 0.20	2.17 ± 0.22	2.06 ± 0.23	2.16 ± 0.23	<0.001
Phosphorus (mmol/L)^a^	1.54 ± 0.46	1.57 ± 0.48	1.43 ± 0.48	1.51 ± 0.48	0.001
Albumin (g/L)^a^	39.1 ± 4.7	34.5 ± 4.3	27.4 ± 4.9	33.7 ± 6.6	<0.001
Prealbumin (g/L)^a^	0.34 ± 0.09	0.30 ± 0.09	0.24 ± 0.10	0.29 ± 0.10	<0.001
CRP (mg/L)^a^	6.94 ± 5.45	8.46 ± 12.47	27.46 ± 44.91	14.28 ± 28.63	<0.001
CAR (×10^-3^)^a^	0.16 (0.13, 0.18)	0.18 (0.15, 0.20)	0.69 (0.19, 1.19)	0.19 (0.15, 0.23)	<0.001
iPTH (pg/ml)^a^	263 ± 201	266 ± 255	217 ± 238	248 ± 233	0.023
Total bilirubin (μmol/L)^a^	7.35 ± 2.74	7.04 ± 3.82	6.57 ± 3.11	6.99 ± 3.27	0.021
Cholesterol (mmol/L)^a^	4.90 ± 1.29	4.74 ± 1.27	4.61 ± 1.16	4.75 ± 1.25	0.031
Triglyceride (mmol/L)^a^	1.86 ± 1.09	1.90 ± 1.56	1.56 ± 0.96	1.78 ± 1.24	0.002
LDL (mmol/L)	2.86 ± 0.93	2.77 ± 1.10	2.75 ± 1.00	2.80 ± 1.01	0.366
HDL (mmol/L)	1.19 ± 0.46	1.14 ± 0.49	1.17 ± 0.53	1.17 ± 0.49	0.51
Blood glucose (mmol/L)	5.39 ± 1.46	5.34 ± 1.69	5.56 ± 2.35	4.53 ± 1.87	0.343
K^+^(mmol/L)^a^	4.12 ± 0.70	3.99 ± 0.79	3.86 ± 0.81	3.99 ± 0.78	0.001

Data are expressed as mean (SD) for Normally distributed continuous variables, median (interquartile range) for Non normally distributed variables, or count (percentage) for categorical variables.

Abbreviation: WBC: white blood cell; NLR: neutrophil to lymphocyte ratio; PLR: platelet to lymphocyte ratio; SCR: serum creatinine; BUN: blood urea nitrogen; CRP: C-reactive protein; CAR:C-reactive protein to albumin; iPTH: intact parathyroid hormone; LDL: low density lipoprotein; HDL: high density lipoprotein.

### 3.2. Survival analysis and Cox regression analysis

**Figure 2.**
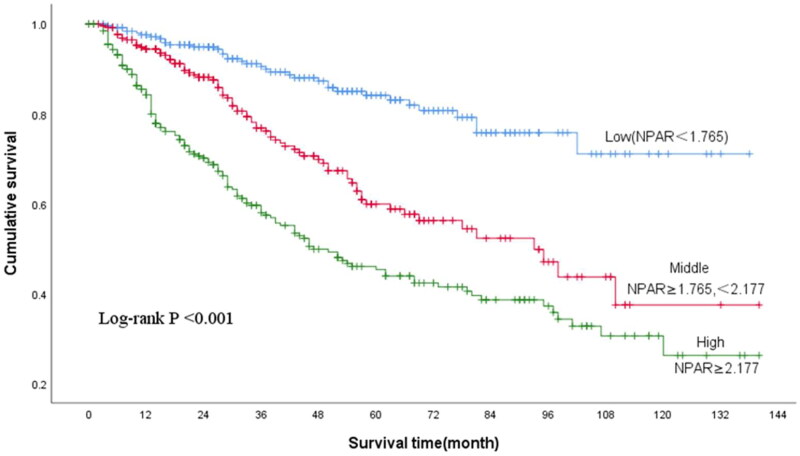
Kaplan Meier curves for overall survival of patients grouped according to NPAR. Abbreviation: NPAR: neutrophil percentage-to-albumin ratio.

**Figure 3.**
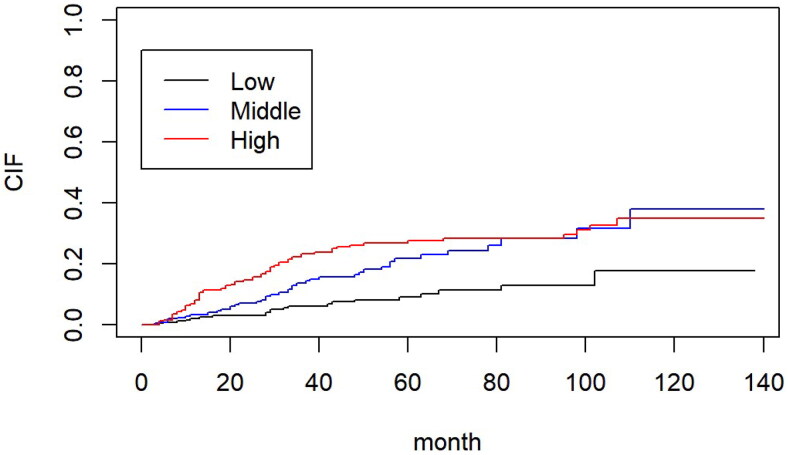
Cumulative incidence of cardio-cerebrovascular death among the groups (Fine-Gray competing risk model, *p* < 0.05).

The average follow-up time was 38.2 (15, 61.5) months. At the end of the follow-up, 243 (30.1%) patients had died, of which 128 (52.7%) had died of cardio-cerebrovascular diseases. The Kaplan–Meier survival curves of the three groups showed that the mortality rates of the middle and high NPAR groups were significantly higher than that of the low NPAR group (*p* < 0.001, Figure 2). The Cox proportional hazards model was used to explore the correlation between different NPAR groups and all-cause mortality in PD patients. Compared with the low NPAR group, the middle and high NPAR groups were associated with an increased risk of all-cause mortality in PD patients (HR: 4.5, 95%; CI: 3.1–6.4, *p* < 0.001), and this result remained statistically significant after adjusting for other cofounders (Model 4, HR: 3.1, 95%; CI: 2.1–4.6, *p* < 0.001), including age, sex, hypertension, diabetes, cardiovascular disease, hemoglobin, platelets, serum creatinine, phosphorus, low density lipoprotein and C-reactive protein (Table 2). The Fine-Grey competing risk model analysis showed that a higher NPAR was associated with a higher cumulative cardio-cerebrovascular mortality rate (SHR = 3.12, *p* < 0.001, Figure 3). After adjusting for some clinical, biochemical, and inflammatory parameters (such as age, sex, hypertension, diabetes and cardiovascular disease, Hb, PLT, SCR, phosphorus, LDL, CRP), the result remained statistically significant (SHR = 2.30, *p* = 0.002) (Table 3).

**Table 2.** Univariate and multivariate analysis of risk factors for all-cause mortality in PD patients.

	Neu (%)		Albumin		NPAR ≥ 1.756, <2.177		NPAR ≥ 2.177	
	HR (95%CI)	*p*-value	HR (95%CI)	*p*-value	HR (95%CI)	*p*-value	HR (95%CI)	*p*-value
Model 1	1.02 (1.00–1.03)	0.008	0.89 (0.87–0.91)	<0.001	2.52 (1.69–3.75)	<0.001	4.46 (3.08–6.45)	<0.001
Model 2	1.02 (1.00–1.03)	0.003	0.90 (0.88–0.92)	<0.001	2.12 (1.42–3.16)	<0.001	3.60 (2.48–5.23)	<0.001
Model 3	1.02 (1.01–1.03)	0.002	0.90 (0.88–0.92)	<0.001	2.16 (1.45–3.23)	<0.001	3.37 (2.30–4.93)	<0.001
Model 4	1.01 (1.00–1.03)	0.050	0.90 (0.88–0.92)	<0.001	2.16 (1.44–3.22)	<0.001	3.13 (2.12–4.62)	<0.001

Model 1: unadjusted.

Model 2: adjust for Age, sex, hypertension, diabetes and cardiovascular disease.

Model 3: adjust for model 2 + hemoglobin, platelet, serum creatinine, phosphorus.

Model 4: adjust for model 3 + low density lipoprotein, C-reactive protein.

Abbreviation: NPAR: neutrophil percentage-to-albumin ratio; Neu (%): neutrophil percentage.

The selected covariates were assessed for multicollinearity using variance inflation factor (VIF) analysis, and no significant multicollinearity was observed among the covariates (VIF < 1.5).

**Table 3.** Association of neu (%), albumin and NPAR with cardio-cerebrovascular death (Fine-Gray competing risk model).

	Neu (%)		Albumin		NPAR ≥ 1.756, <2.177		NPAR ≥ 2.177	
	SHR (95%CI)	*p*-value	SHR (95%CI)	*p*-value	SHR (95%CI)	*p*-value	SHR (95%CI)	*p*-value
Model 1	1.00 (0.97–1.02)	0.95	0.92 (0.89–0.94)	<0.001	2.25 (1.35–3.77)	0.002	3.12 (1.90–5.11)	<0.001
Model 2	1.00 (0.98–1.02)	<0.001	0.92 (0.90–0.95)	<0.001	1.92 (1.14–3.24)	0.01	2.44 (1.48–4.03)	<0.001
Model 3	1.00 (0.98–1.02)	0.95	0.93 (0.90–0.96)	<0.001	1.92 (1.14–3.23)	0.01	2.27 (1.35–3.81)	0.002
Model 4	1.00 (0.98-1.02)	0.91	0.93 (0.90-0.96)	<0.001	1.94 (1.15-3.27)	0.01	2.30 (1.34-3.91)	0.002

Model 1: unadjusted.

Model 2: adjust for Age, sex, hypertension, diabetes and cardiovascular disease.

Model 3: adjust for model 2 + hemoglobin, platelet, serum creatinine, phosphorus.

Model 4: adjust for model 3 + low density lipoprotein, C-reactive protein.

Abbreviation: NPAR: neutrophil percentage-to-albumin ratio; Neu (%): Neutrophil Percentage.

### 3.3. Comparative analysis of prognostic parameters in PD patients

**Figure 4.**
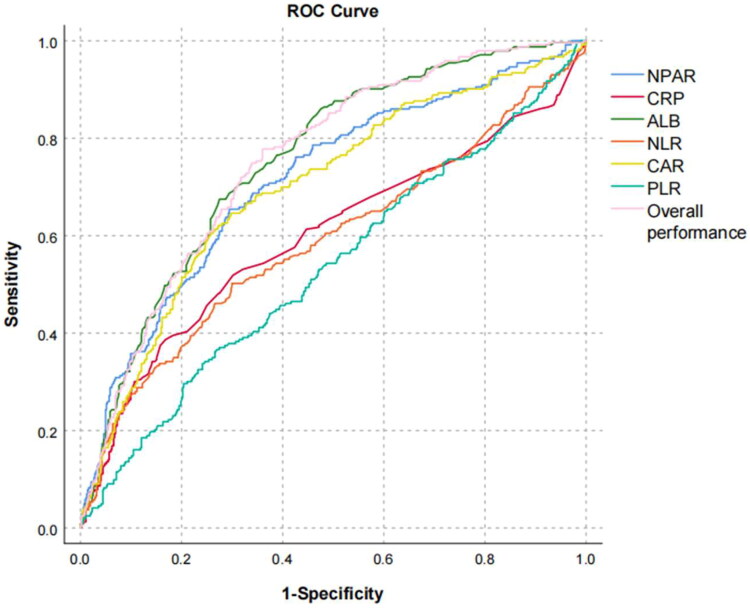
ROC curve of various parameters and overall performance in predicting death in peritoneal dialysis patients. Abbreviation: NPAR: neutrophil percentage-to-albumin ratio; CRP:C-reactive protein, NLR: neutrophil to lymphocyte ratio, CAR: C-reactive protein to albumin ratio, PLR: platelet to lymphocyte ratio

Single-factor ROC analysis of prognostic parameters in PD patients showed that the NPAR had an AUC of 0.714 (95% CI: 0.675–0.754), which was higher than that of CRP (0.597, 95% CI: 0.551–0.644, DeLong *p* value < 0.001), NLR (0.589, 95% CI: 0.543–0.635, DeLong p value < 0.001) and CAR (0.698, 95% CI: 0.658–0.738, DeLong *p* value = 0.642) and lower than that of ALB (0.754, 95% CI: 0.719–0.789, DeLong *p* value < 0.001). The optimal cutoff value of the NPAR was 2.06, with a sensitivity and specificity of 65% and 71%, respectively, indicating that the risk of death in peritoneal dialysis patients increased when the NPAR was greater than 2.06 (Figure 4, Table 4).

**Table 4.** Single factor ROC analysis of NPAR, CRP, albumin, NLR, CAR, PLR and Overall performance.

	AUC	95%CI	*p*-value	Cutoff	DeLong *p*-value	Sensitivity	Specificity	D-stat	R^2^
NPAR	0.714	0.675–0.754	<0.001	2.06		0.65	0.71	0.428	0.150
CRP	0.597	0.551–0.644	<0.001	7.35	<0.001	0.39	0.83	0.195	0.032
Albumin	0.754	0.719–0.789	<0.001	32.15	0.005	0.72	0.68	0.508	0.212
NLR	0.589	0.543–0.635	<0.001	3.58	<0.001	0.50	0.70	0.178	0.018
CAR	0.698	0.658–0.738	<0.001	0.192	0.462	0.65	0.70	0.396	0.048
PLR	0.533	0.488–0.578	0.147	211.05	<0.001	0.34	0.76	0.066	0.005
Overall	0.755	0.719–0.790	<0.001	–	<0.001	0.72	0.68	0.509	0.175

Abbreviation: NPAR: NPAR: neutrophil percentage-to-albumin ratio; CRP: C-reactive protein; NLR: neutrophil to lymphocyte ratio; CAR:C-reactive protein to albumin; PLR: platelet to lymphocyte ratio.

**Figure 5.**
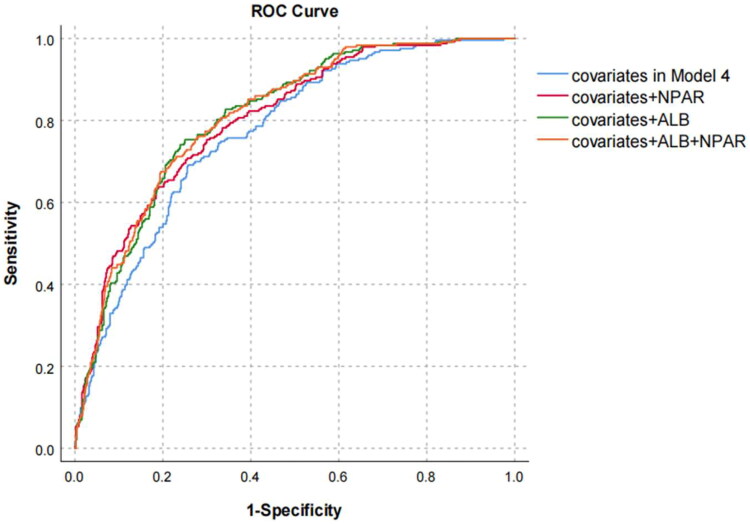
ROC curve based on multifactor analysis. Abbreviation: covariates: age, sex, hypertension, diabetes, cardiovascular disease, hemoglobin, platelet, serum creatinine, phosphorus, low density lipoprotein and C-reactive protein; NPAR: neutrophil percentage-to-albumin ratio. ALB: albumin.

The ROC curve based on Model 4 was plotted to explore the impact of ALB and NPAR on outcome in the multivariable analysis. The model comprising covariates (age, sex, hypertension, diabetes, cardiovascular disease, hemoglobin, platelets, serum creatinine, phosphorus, low-density lipoprotein and C-reactive protein) yielded an AUC of 0.771 (95% CI: 0.737–0.805, *p* < 0.001). When ALB and NPAR were added to covariates, there was a significant improvement in prediction performance (AUC = 0.809; 95% CI: 0.779–0.840, AUC = 0.802; 95% CI: 0.770–0.834). When both ALB and NPAR were included together, there was a slight improvement in the model (AUC = 0.811; 95% CI: 0.780–0.841) (Figure 5, Table 5).

**Table 5.** Multifactor ROC analysis.

	AUC	95%CI	*p*-value	D-stat	R^2^
Covariates	0.771	0.737–0.805	<0.001	0.542	0.261
Covariates + NPAR	0.802	0.770–0.834	<0.001	0.604	0.314
Covariates + albumin	0.809	0.779–0.840	<0.001	0.618	0.343
Covariates + albumin + NPAR	0.811	0.780–0.841	<0.001	0.622	0.343

Abbreviation: Covariates: age, sex, hypertension, diabetes, cardiovascular disease, hemoglobin, platelet, serum creatinine, phosphorus, low density lipoprotein and C-reactive protein; NPAR: neutrophil percentage-to-albumin ratio.

**Figure 6.**
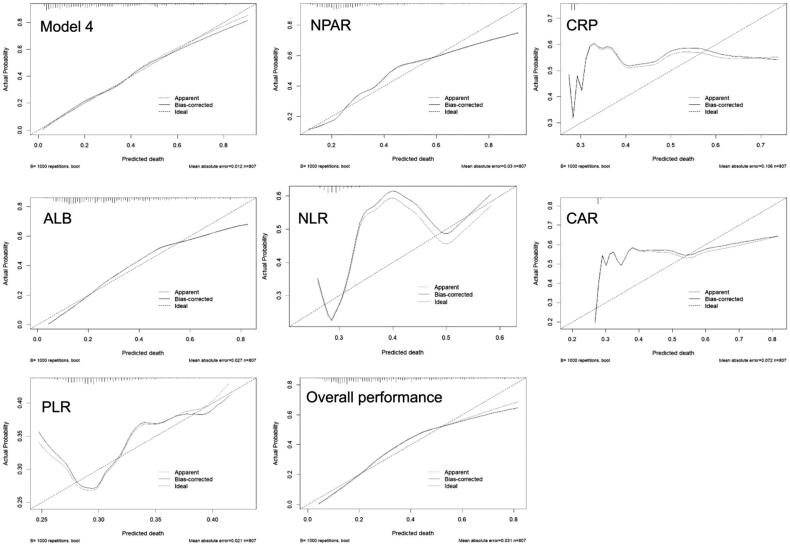
Calibration plot of model 4, NPAR, CRP, ALB, NLR, CAR, PLR and Overall performance. Abbreviation: NPAR: neutrophil percentage-to-albumin ratio; CRP: C-reactive protein; ALB: albumin; NLR: neutrophil to lymphocyte ratio; CAR:C-reactive protein to albumin; PLR: platelet to lymphocyte ratio.

The calibration plots revealed that the curve of Model 4 generally lay close to the 45-degree line. In univariate prediction models, NPAR and ALB were closer to the ideal curve, while for higher risk levels (predicted probability >0.6), the curves began to deviate downwards. On the other hand, there was a significant discrepancy between predicted probabilities and actual probabilities for CRP, NLR, CAR and PLR (Figure 6).

## 4. Discussion

The current study revealed that elevated NPAR was strongly associated with increased all-cause mortality and cardio-cerebrovascular mortality in PD patients, independent of clinical, biochemical, and inflammatory parameters. In addition, the AUC of the NPAR in predicting the risk of all-cause mortality in PD patients was significantly higher than those of CRP, NLR, CAR and PLR, but slightly lower than that of ALB. To the best of our knowledge, this is the first study indicating that the NPAR can be used as a parameter reflecting adverse outcomes in PD patients.

Inflammation and malnutrition are two important risk factors for mortality in patients with PD [14,15]. Neutrophils and albumin are two common clinical indicators used to evaluate inflammation and nutritional status in PD patients. Peritoneal dialysate stimulation, infection, endotoxin and cytokine induction, loss of residual renal function, chronic heart failure and fluid retention are common causes of chronic inflammation [4]. The chronic inflammatory state may lead to peritoneal fibrosis [16], atherosclerosis deterioration and cardiovascular disease [17], which negatively affect the prognosis of PD patients. Moreover, inflammation and malnutrition can affect each other to form a vicious cycle in PD patients. For example, PD-related peritonitis may lead to substantial loss of protein in PD fluid, resulting in hypoalbuminaemia and malnutrition, which have been found to be associated with the loss of residual kidney function [18], infection, and increased mortality [19]. Alternatively, albumin can stimulate neutrophils through voltage-gated proton channels to produce reactive oxygen species (ROS) and elastase, exacerbating inflammatory states [20]. Therefore, inflammation and malnutrition both affect the death of PD patients, and a single prognostic marker cannot fully account for the high mortality rate of ESRD patients.

NPAR refers to the ratio of neutrophil percentage count to albumin level, reflecting both the inflammatory and nutritional states of patients, which may better reflect the adverse outcome of PD than a single marker. Such a hypothesis has been confirmed in many nonrenal diseases. For example, many studies suggest that the NPAR may predict poor outcomes in patients with various types of cancer, and its predictive capacity is better than that of neutrophil percentage or albumin alone [7]. On the other hand, high levels of NPAR have been shown as an independent risk factor for all-cause mortality in patients with various cardio-cerebrovascular diseases, such as atrial fibrillation, coronary heart disease, heart failure [21], and stroke [10]. Despite this evidence, the relationship between the NPAR and long-term PD patients is not clear. We found that the all-cause mortality rates of the medium and high NPAR groups were significantly higher than those of the low NPAR group (HR: 2.52, 95% CI: 1.69–3.75, *p* < 0.00, and HR: 4.46, 95% CI: 3.08–6.45, *p* < 0.001). This result was still valid after adjusting for confounders, including demographic data, anemia, lipid and mineral parameters. This suggested that the NPAR was an independent risk factor for all-cause mortality in PD patients.

As the principal cause of death in PD patients is cardio-cerebrovascular disease [22,23], we further explored the relationship between the NPAR and cardio-cerebrovascular mortality using a competing risk model (CRM). The CRM showed that the cumulative incidence of cardio-cerebrovascular mortality was significantly higher in the middle and high NPAR groups than in the low NPAR group (Figure 3), with the risk of cardiovascular and cerebrovascular mortality in the high NPAR group being 2.07 times higher than that in the low NPAR group. Noteworthy, neutrophil percentage (Neu%) and albumin were both related to cardio-cerebrovascular mortality.

CRP, NLR, ALB are traditional markers used to evaluate the risk of death in PD patients. Nan Yang et al. pointed out in their review that NLR has a good predictive ability for the complications in dialysis patients, including CVD and infection [24]. Previous research has shown that the Pan-immune-inflammation value (PIV) is also associated with all-cause death, CVD and infection in PD patients [25]. Recently, the PLR and CAR have been used to predict the prognosis of PD patients [26]. Our study compared the predictive capacity of NPAR with some these markers and revealed that NPAR had a higher predictive value as an independent risk factor for all-cause mortality in PD patients, with the AUC of ROC higher than those of CRP, NLR and CAR but lower than that of ALB, while PLR was not able to predict the adverse outcome of PD patients (*p* = 0.147). Many studies have verified that composite indicators are superior to a single indicator in assessing the prognosis of patients (such as cancers and critically ill patients). However, the AUC value of the NPAR was lower than that of ALB in our study, indicating that hypoalbuminaemia may have a greater impact on mortality in peritoneal dialysis than chronic inflammation. We further plotted the ROC curve based on Model 4 and found that the AUC was 0.771. Sequentially adding ALB and NPAR could further enhance the predictive performance (AUC = 0.809, AUC = 0.811) (Figure 5, Table 5). In addition, our calibration plots (Figure 6) showed that the curve of Model 4 was the closest to the ideal 45-degree line. This suggests that Model 4 provides a relatively accurate prediction of all-cause mortality in PD patients. In univariate prediction models, NPAR and ALB are closer to the 45-degree line. However, for higher risk levels (predicted death probability >0.6), the curves begin to deviate downwards. Alternatively, we observed a significant deviation for the curves of CRP, NLR, CAR and PLR. This indicates that these indicators alone may not accurately predict the outcome, and that their predictive value and accuracy were inferior to that of the NPAR. The optimal cutoff value of the NPAR was 2.06, indicating that when the NPAR value was greater than 2.06, the mortality rate of PD patients increased.

This study, to our knowledge, pioneered the investigation of the correlation of the NPAR with poor outcomes in PD patients. It also uniquely compared to the predictive capacities of the NPAR, CRP, NLR, CAR, and PLR. The strengths of this study include a ten-year follow-up period, a large sample size, and a low drop-out rate of the patients. There are also some limitations of this study. First, this is a single-center, retrospective study with the possibility of recall bias, and we only collected data from a single time point of NPAR, without further studying the change in NPAR over time. Second, residual renal function, dialysis adequacy, cardiovascular events, fibrinogen and erythrocyte sedimentation rate and other nutrition-related indicators, such as subjective global assessment (SGA), were not analyzed in this study. Last, we did not exclude the influence of drugs on the research variables. Therefore, multicenter, prospective longitudinal studies are still needed to further verify our results in the future.

## 5. Conclusion

The NPAR can be used as an effective independent predictive indicator for all-cause mortality risk and cardio-cerebrovascular mortality in long-term PD patients. The correlation between the NPAR and poor prognosis in PD patients was higher than those of CRP, CAR, NLR, and PLR. Therefore, monitoring the NPAR level was useful to identify high-risk PD patients.

## Ethical approval

This study was approved by the Ethics Committee of the Second Affiliated Hospital of Soochow University. (Approval No.: JD-LK-2021-002-01).

## Supplementary Material

Supplemental Material

Supplemental Material

## References

[1] Yu X, Yang X, Huang N. Management of a rapidly growing peritoneal dialysis population at the first affiliated hospital of Sun Yat-Sen University. Perit Dial Int. 2014;34 Suppl 2(Suppl 2):1–9. doi: 10.3747/pdi.2013.00122. PMC4076964 24962960

[2] Li PK, Chow KM, Van de Luijtgaarden MW, et al. Changes in the worldwide epidemiology of peritoneal dialysis. Nat Rev Nephrol. 2017;13(2):90–103. doi: 10.1038/nrneph.2016.181. 28029154

[3] Jegatheesan D, Cho Y, Johnson DW. Clinical studies of interventions to mitigate cardiovascular risk in peritoneal dialysis patients. Semin Nephrol. 2018;38(3):277–290. doi: 10.1016/j.semnephrol.2018.02.007. 29753403

[4] Wang AY. Consequences of chronic inflammation in peritoneal dialysis. Semin Nephrol. 2011;31(2):159–171. doi: 10.1016/j.semnephrol.2011.01.005. 21439430

[5] Han SH, Han DS. Nutrition in patients on peritoneal dialysis. Nat Rev Nephrol. 2012;8(3):163–175. doi: 10.1038/nrneph.2012.12. 22310948

[6] Kendrick J, Chonchol MB. Nontraditional risk factors for cardiovascular disease in patients with chronic kidney disease. Nat Clin Pract Nephrol. 2008;4(12):672–681. doi: 10.1038/ncpneph0954. 18825155

[7] Ko CA, Fang KH, Tsai MS, et al. Prognostic value of neutrophil percentage-to-albumin ratio in patients with oral cavity cancer. Cancers. 2022;14(19):4892. doi: 10.3390/cancers14194892. PMC9564168 36230814

[8] Wang X, Wang J, Wu S, et al. Association between the neutrophil percentage-to-albumin ratio and outcomes in cardiac intensive care unit patients. Int J Gen Med. 2021;14:4933–4943. doi: 10.2147/IJGM.S328882. 34483683 PMC8409768

[9] Sun T, Shen H, Guo Q, et al. Association between neutrophil percentage-to-albumin ratio and all-cause mortality in critically ill patients with coronary artery disease. Biomed Res Int. 2020;2020:8137576. doi: 10.1155/2020/8137576. 32934964 PMC7479485

[10] Cui T, Wang C, Zhu Q, et al. Association between neutrophil percentage-to-albumin ratio and 3-month functional outcome in acute ischemic stroke patients with reperfusion therapy. Front Neurol. 2022;13:898226. doi: 10.3389/fneur.2022.898226. 36176549 PMC9513151

[11] Wang X, Han Q, Wang T, et al. Serum albumin changes and mortality risk of peritoneal dialysis patients. Int Urol Nephrol. 2020;52(3):565–571. doi: 10.1007/s11255-020-02389-y. 32016905

[12] Lu X, Wang S, Zhang G, et al. High neutrophil-to-lymphocyte ratio is a significant predictor of cardiovascular and all-cause mortality in patients undergoing peritoneal dialysis. Kidney Blood Press Res. 2018;43(2):490–499. doi: 10.1159/000488696. 29627842

[13] Chen T, Yang M. Platelet-to-lymphocyte ratio is associated with cardiovascular disease in continuous ambulatory peritoneal dialysis patients. Int Immunopharmacol. 2020;78:106063. doi: 10.1016/j.intimp.2019.106063. 31835088

[14] Chen TK, Knicely DH, Grams ME. Chronic kidney disease diagnosis and management: a review. JAMA. 2019;322(13):1294–1304. doi: 10.1001/jama.2019.14745. 31573641 PMC7015670

[15] Nusair MB, Rajpurohit N, Alpert MA. Chronic inflammation and coronary atherosclerosis in patients with end-stage renal disease. Cardiorenal Med. 2012;2(2):117–124. doi: 10.1159/000337082. 22851960 PMC3376340

[16] Zhou Q, Bajo MA, del Peso G, et al. Preventing peritoneal membrane fibrosis in peritoneal dialysis patients. Kidney Int. 2016;90(3):515–524. doi: 10.1016/j.kint.2016.03.040. 27282936

[17] Ortiz A, Covic A, Fliser D, E.-, et al. Board of the, epidemiology, contributors to, and clinical trials of mortality risk in chronic kidney failure. Lancet. 2014;383(9931):1831–1843. doi: 10.1016/S0140-6736(14)60384-6. 24856028

[18] Yamada S, Kawai Y, Tsuneyoshi S, et al. Lower serum albumin level is associated with an increased risk for loss of residual kidney function in patients receiving peritoneal dialysis. Ther Apher Dial. 2020;24(1):72–80. doi: 10.1111/1744-9987.12861. 31125508

[19] Kiebalo T, Holotka J, Habura I, et al. Nutritional status in peritoneal dialysis: nutritional guidelines, adequacy and the management of malnutrition. Nutrients. 2020;12(6):1715. doi: 10.3390/nu12061715. 32521626 PMC7352713

[20] Zhao R, Dai H, Arias RJ, et al. Direct activation of the proton channel by albumin leads to human sperm capacitation and sustained release of inflammatory mediators by neutrophils. Nat Commun. 2021;12(1):3855. doi: 10.1038/s41467-021-24145-1. 34158477 PMC8219737

[21] Hu Z, Wang J, Xue Y, et al. The neutrophil-to-albumin ratio as a new predictor of all-cause mortality in patients with heart failure. J Inflamm Res. 2022;15:701–713. doi: 10.2147/JIR.S349996. 35140500 PMC8818978

[22] Miglinas M, Cesniene U, Janusaite MM, et al. Cerebrovascular disease and cognition in chronic kidney disease patients. Front Cardiovasc Med. 2020;7:96. doi: 10.3389/fcvm.2020.00096. 32582768 PMC7283453

[23] Krediet RT, Balafa O. Cardiovascular risk in the peritoneal dialysis patient. Nat Rev Nephrol. 2010;6(8):451–460. doi: 10.1038/nrneph.2010.68. 20567248

[24] Yang N, Yang K, Pan S, et al. Progress in the application of the neutrophil-to-lymphocyte ratio in dialysis-related complications. Ren Fail. 2023;45(2):2259996. 37791567 10.1080/0886022X.2023.2259996 PMC10552595

[25] Zhang F, Li L, Wu X, et al. Pan-immune-inflammation value is associated with poor prognosis in patients ­undergoing peritoneal dialysis. Ren Fail. 2023;45(1):2158103. 36632816 10.1080/0886022X.2022.2158103 PMC9848369

[26] Su N, Zheng Y, Zhang X, et al. Platelet-to-lymphocyte ratio and the first occurrence of peritonitis in peritoneal dialysis patients. BMC Nephrol. 2022;23(1):415. doi: 10.1186/s12882-022-03038-5. 36585653 PMC9803258

