# Supplementary material for: The neutrophil percentage-to-albumin ratio is an independent risk factor for poor prognosis in peritoneal dialysis patients
Source: Ren Fail. 2024 Jan 4;46(1):2294149. doi: 10.1080/0886022X.2023.2294149 (PMC10773631; doi:10.1080/0886022X.2023.2294149)
Supplement: Supplemental Material [file IRNF_A_2294149_SM4869.pdf]

Figure s1: Schoenfeld Residual Test for Model 4

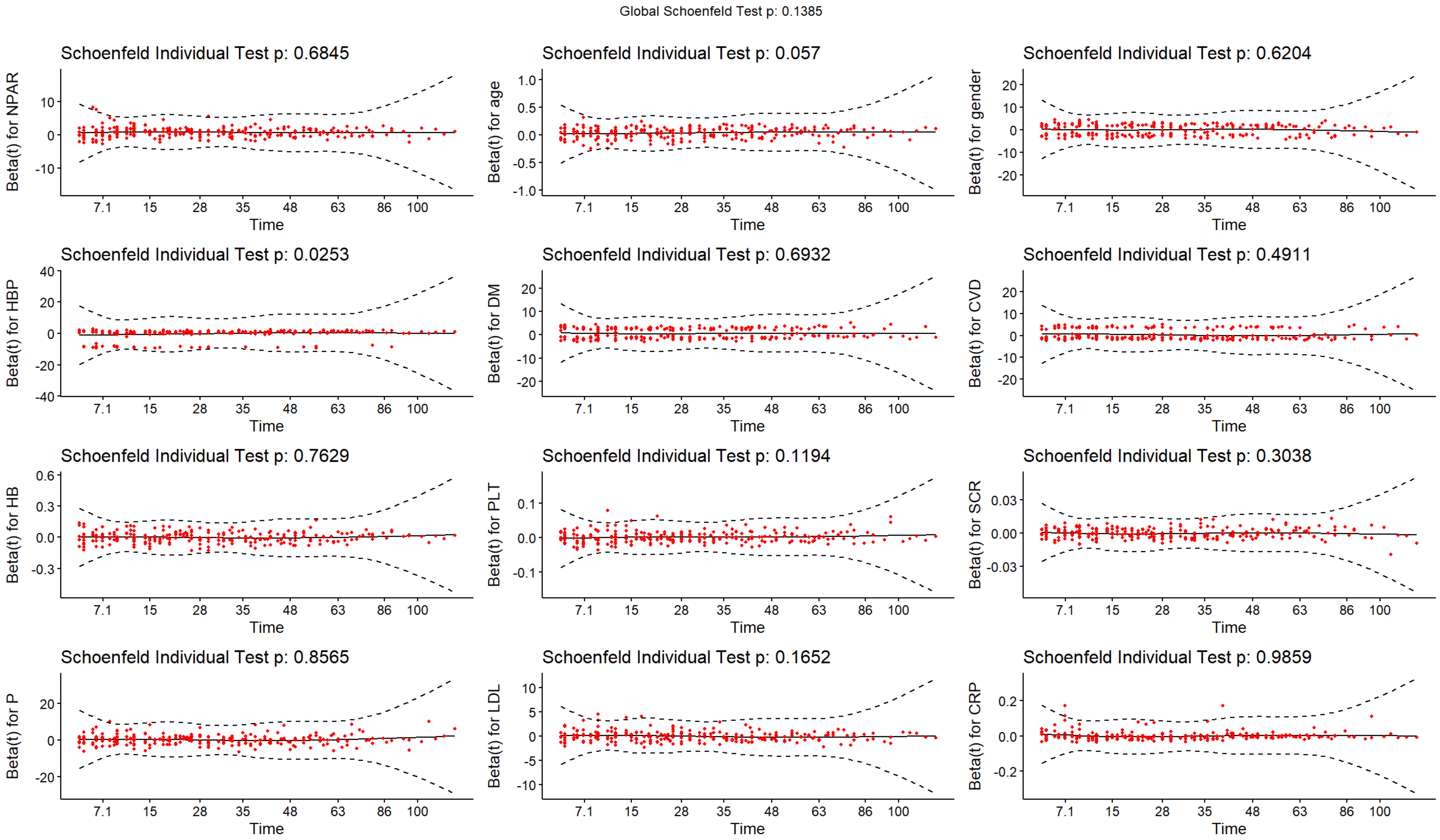

Information Classification: General

Figure s2: Restricted Cubic Spline for the neutrophil percentage-to-albumin ratio

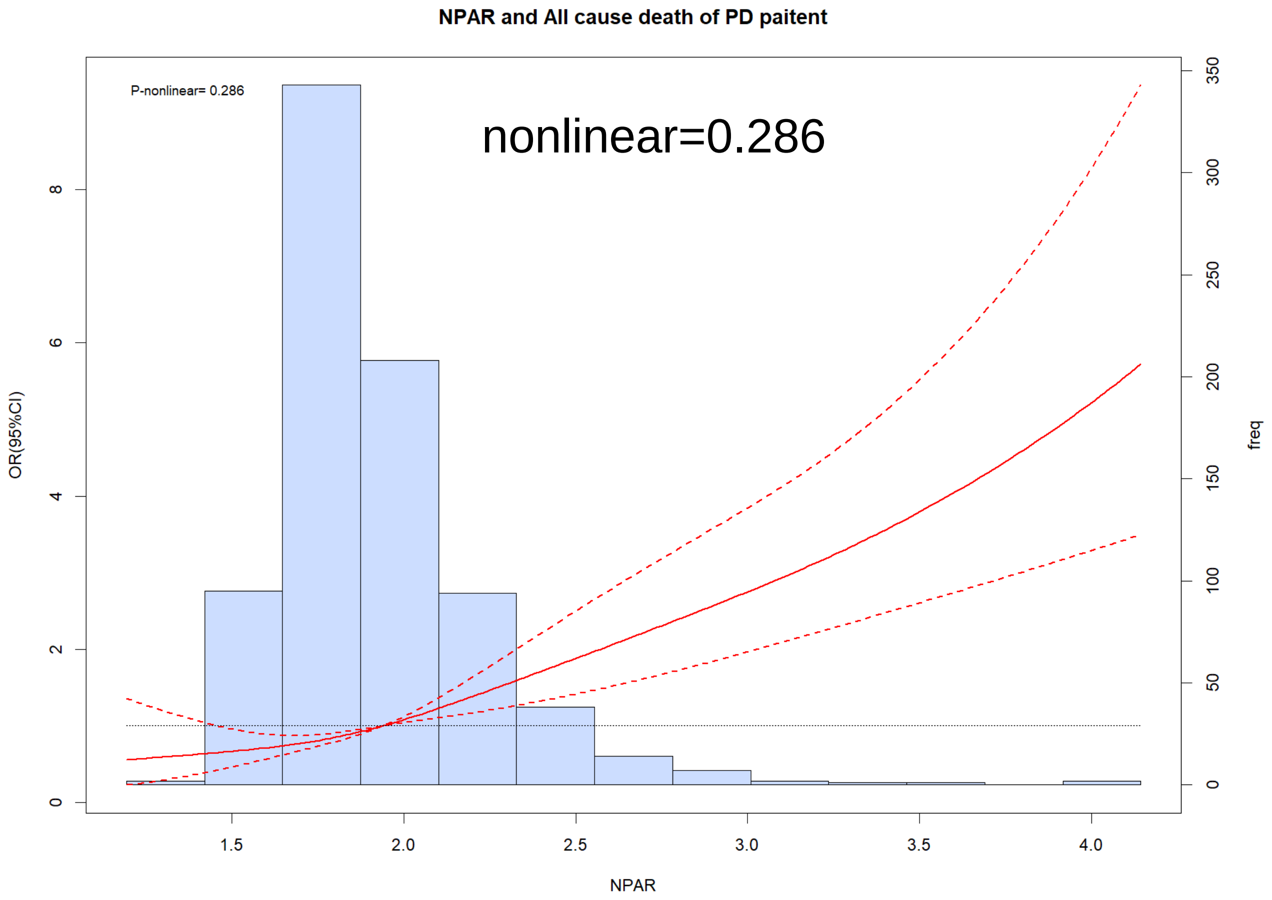

Information Classification: General

Figure s3: calibration plot of Cox proportional hazard model 4

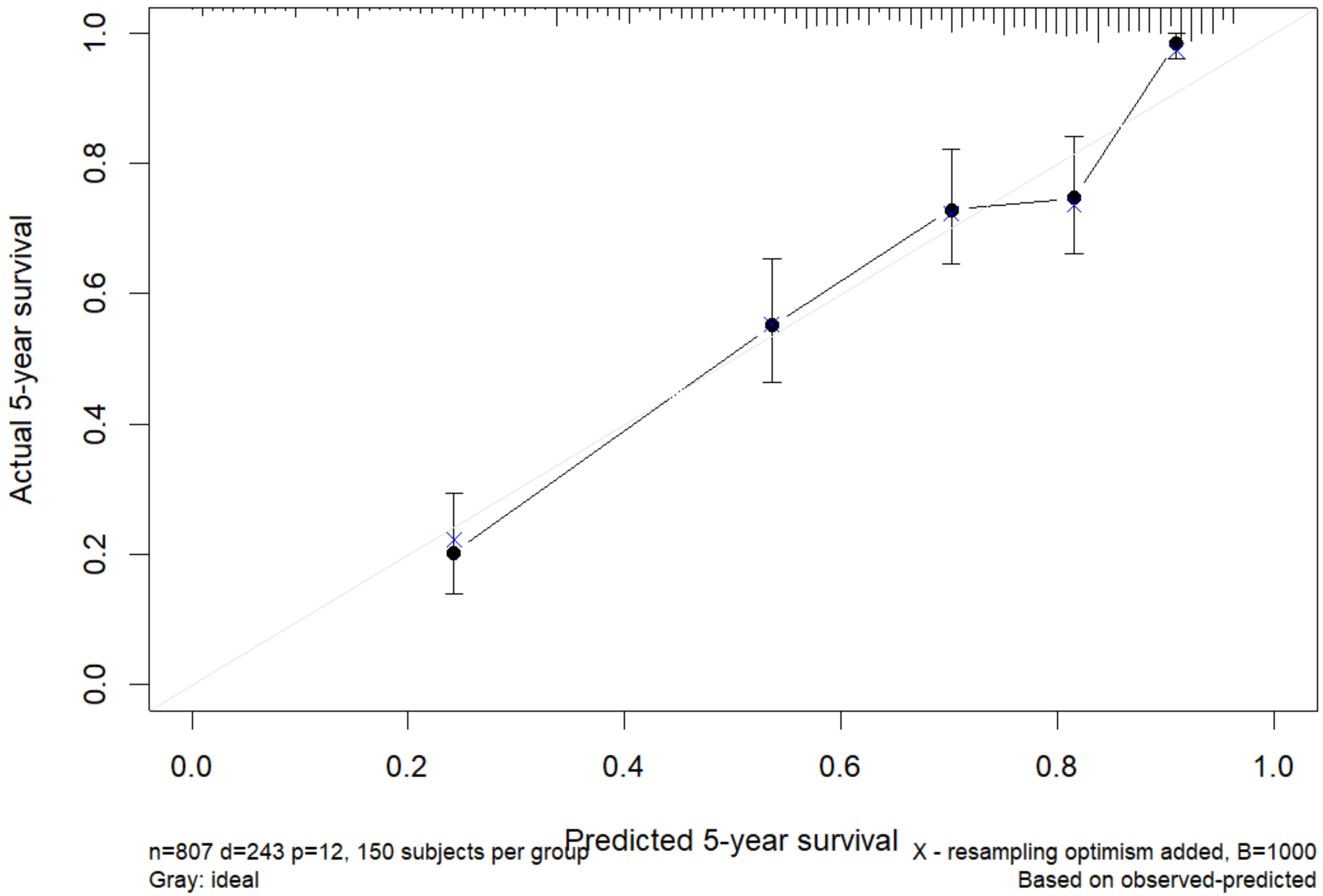

Information Classification: General

Figure s4: Predicted Survival Curve of Cox proportional hazard model 4

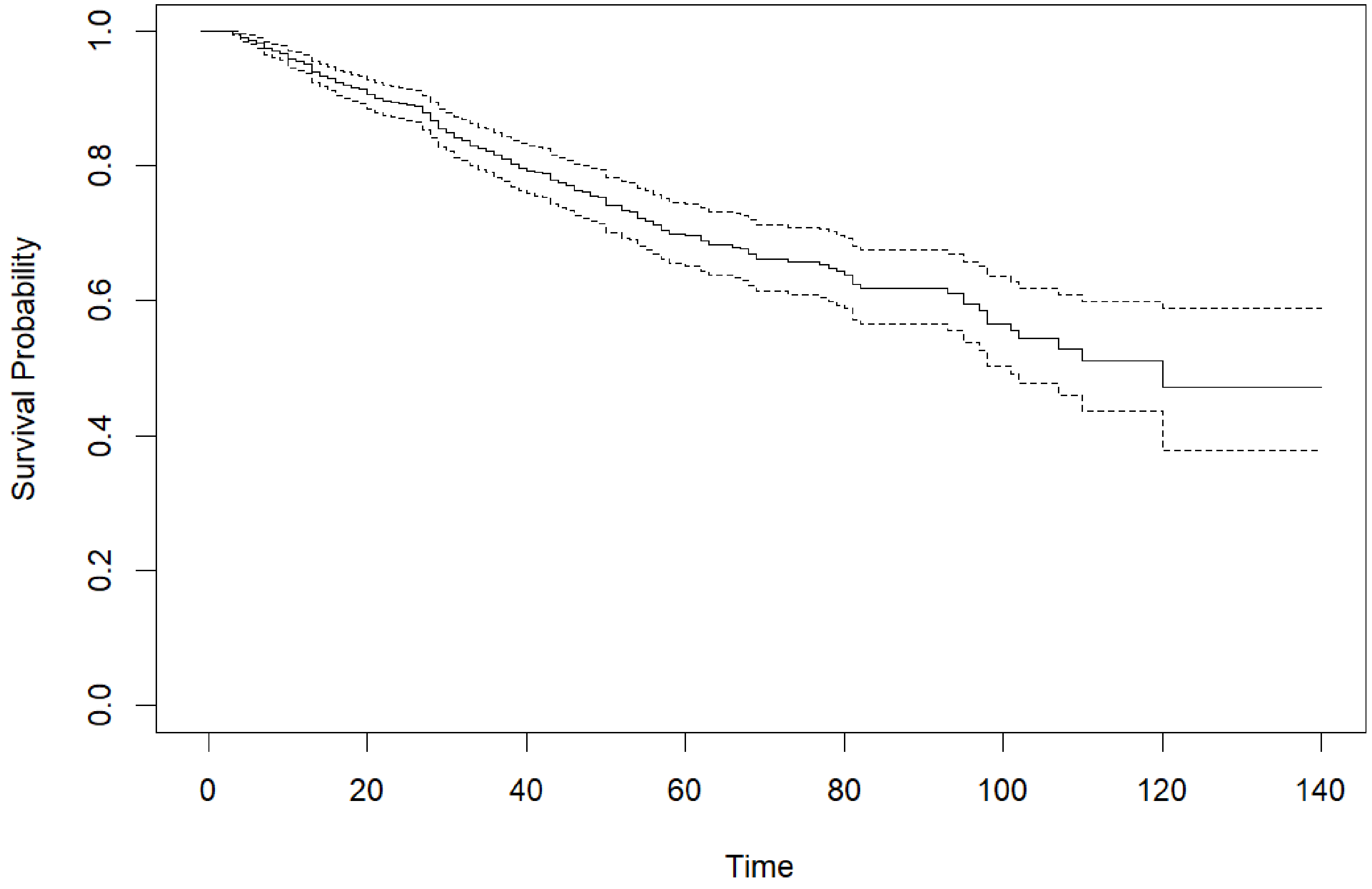

Information Classification: General

Table s1: Hosmer-Lemeshow statistic

	X−squared	df	Hosmer-Lemeshow statistic
Model 4	16.8	12	0.158
NPAR	3.76	1	0.052
CRP	14.58	1	＜0.001
ALB	1.33	1	0.248
NLR	16.89	1	＜0.001
CAR	48.2	1	＜0.001
PLR	0.411	1	0.521
Overall	7.38	6	0.287

Information Classification: General
